# In utero xenotransplantation of mice bone marrow-derived stromal/stem cells into fetal rat liver: An experimental study

**DOI:** 10.18502/ijrm.v13i9.7665

**Published:** 2020-09-20

**Authors:** Maryam Kasraeian, Elahe Ghasemi, Mehdi Dianatpour, Nader Tanideh, Iman Jahromi Razeghian, Zahra Khodabandeh, Mohammad Reza Dorvash, Shahrokh Zare, Omid Koohi Hosseinabadi, Amin Tamadon

**Affiliations:** ^1^Department of Obstetrics and Gynecology, Maternal-Fetal Medicine Research Center, Perinatology Ward, Shiraz University of Medical Sciences, Shiraz, Iran.; ^2^Stem Cells Technology Research Center, Shiraz University of Medical Sciences, Shiraz, Iran.; ^3^Department of Human Genetic, School of Medicine, Shiraz University of Medical Sciences, Shiraz, Iran.; ^4^Department of Pharmacology, School of Medicine, Shiraz University of Medical Sciences, Shiraz, Iran.; ^5^Cardiovascular Research Center, Shiraz University of Medical Sciences, Shiraz, Iran.; ^6^Central Lab, Shiraz University of Medical Sciences, Shiraz, Iran.; ^7^The Persian Gulf Marine Biotechnology Research Center, the Persian Gulf Biomedical Sciences Research Institute, Bushehr University of Medical Sciences, Bushehr, Iran.

**Keywords:** Xenotransplantation, Liver, Bone marrow, Stromal/stem cell, Murine

## Abstract

**Background:**

Animals can play an important role in preparing tissues for human through the development of xenotransplantation protocols. The most common problem with liver transplantation like any other organ transplantation is organ supply shortage.

**Objective:**

To evaluate the in utero xenotransplantation of mouse bone marrow-derived stromal/stem cells (BMSCs) to the liver of rat fetus to produce mouse liver tissue.

**Materials and Methods:**

BMSCs were isolated and confirmed from enhanced green fluorescent protein (eGFP)-genetic labeled mice. Using a microinjection protocol, mice BMSCs were injected into the liver of rat fetuses in utero on day 14 of pregnancy. After birth, livers were collected and the presence of mice eGFP-positive cells in rat livers was evaluated through polymerase chain reaction.

**Results:**

The eGFP mRNA was detected in the liver of injected infant rats. BMSCs of adult mice were capable to remain functional probably as hepatocyte-like cells in liver of infant rats after in utero xenotransplantation.

**Conclusion:**

BMSCs have the potential for intrauterine xenotransplantation for the treatment of liver dysfunction before birth. This method can also be used for xenoproduction of liver tissue for transplantation.

## 1. Introduction

Stromal/stem cells (SCs) are known as undifferentiated cells, with the ability to either divide into identical daughter cells (proliferative capability and self-renewal) or to differentiate into specialized somatic cells (differentiation potential) (1). These cells were identified and isolated from different type of tissues (2-7). The SCs in bone marrow stroma comprise a restricted area; however, it can be easily proliferated. These cells can be used for the treatment of non-hematopoietic diseases (8-10).

Liver cirrhosis indicated via regenerative nodules' formation and hepatic architecture impairment is a late phase of progressive hepatic fibrosis (11). It is generally irreversible in its advanced stages. Liver transplantation has long been the unique curative treatment for acute or chronic liver diseases (12). It involves several essential issues like surgical difficulties, lack of donors, rejection, and high value of expenses.

Owing to the many advances in prenatal diagnosis and microsurgery techniques, intervention at fetal period has become one of the most advanced techniques to treat or prevent many deadly diseases that begin or are diagnosed at the early stages of embryologic development (13). Prenatal treatment can prevent from many after-birth complications due to the fact that some genetic diseases may damage organs in utero irreparably (14). Therefore, stem cell transplantation in a living organism is considered as an important clinical measure in managing the patients with congenital, anemia, metabolic, and immune system abnormalities (15). Xenotransplantation means producing an organ from one species in other species using transplantation method. Studies have been performed on in utero injection of human or animal stem cells using sheep and pigs as the host and the cells have been injected into the cavity of peritoneum, heart or liver of the fetuses (16). The ease of separation and proliferation of mice bone marrow-derived stromal/stem cells (BMSCs) made it a good choice for our study to inject into fetus of rat before formation of fetal immune system.

Considering the time of immune system formation in the fetus, Table I displays different mammals that can be used for in utero xenotransplantation. In the early stages of pregnancy, since the fetal immune system is not complete, it cannot recognize foreign tissues, and therefore the xenotransplanted cells are not rejected. In the present investigation, we decided to examine intrauterine injection of BMSCs in mice with enhanced green fluorescent protein (eGFP) into the rat fetal liver and also investigate the presence of *GFP* gene in rat pup's liver.

**Table 1:** Characters of animal species that can be applied as recipient of human tissue by xenotransplantation


**Species**	**Fetal commencement of immune system**	**Pregnancy duration**	**Fetus per delivery**	**Human tissue or cell xenotransplantation**
**Monkey**	Day 35 (17)	164 days	1-2	CB-SCs (18)
**Cow**	Day 40 (19)	284 days	1	ND
**Camel**	Day 90 (20)	390 days	1	ND
**Buffalo**	ND	315 days	1	ND
**Pig**	Day 17	114 days	8-10	BMSCs (21)
**Sheep**	Days 64-82 (19)	148 days	1-3	SCs (16)
**Goat**	Days 64-82 (19)	150 days	2	ATSCs (22)
**Dog**	Day 27-45 (19)	60 day	6-10	ND
**Rabbit**	Day 14 (23)	33 days	4-8	ATSCs (24)
**Guinea pig**	Day 40-47 (25)	59-72 days	3-12	BMSCs (26)
**Rat**	Day 14 (27)	22 days	10-12	ATSCs and BMSCs (28)
**Mouse**	Day 12 (27)	20 days	10-12	BMSCs (29)
ATSCs: Adipose tissue-derived stromal/stem cells; BMSCs: Bone marrow-derived stromal/stem cells; CB-SCs: Cord blood stem cells; ND: No available data; SCs, stem cells				

## 2. Materials and Methods

### Animals

For this experimental study, Three adult female (12 weeks, 250 ± 30 gr) and three male (10 weeks, 280 ± 10 gr) Sprague-Dawley rats were purchased from the Comparative and Experimental Medicine Center of Shiraz University of Medical Sciences, Shiraz, Iran and two male eGFP${}^{+/+}$ Balb/c mice were gifted by the Royan Institute, Tehran, Iran. The animals were housed under controlled temperature (23 ± 1°C), 55 ± 5% relative humidity, and 12 hr light/dark cycle. They had free access to standard chow and water during the experimental period.

### BMSCs isolation

The adult male eGFP mice were euthanized by ether for isolation of bone-marrow stromal/stem cells. In details, after disinfecting the entire body by ethanol and removing the humerus and tibia bones from surrounding muscles, the mice were placed in Falcon tubes containing phosphate buffered saline (PBS, Gibco, USA) and penicillin-streptomycin antibiotics (Sigma, USA). Under the class II cell culture hood, both ends of the bones were cut using scissors, and the bone marrow canal was flushed using a needle connected to a syringe containing Dulbecco's Modified Eagle's medium (DMEM, Gibco, USA). The cell suspension was collected in a Falcon tube and was then centrifuged at 1200 rpm for 5 min. After removing the supernatant, 1 mL cell culture medium was added to the sediment and cells were re-suspended by pipetting. Complete culture medium contained 90% DMEM (low glucose, Gibco, USA), 10% bovine calf serum (FBS, Gibco, USA), 1% penicillin-streptomycin antibiotic (Sigma), 1% antifungal solution (Sigma, USA), and 1% non-essential amino acid (Sigma, USA). The cell mixture was transferred to a 25T flask containing 4 mL of complete culture medium and incubated in 5% CO${}_{2}$ at 37°C. After 24 hr, 5 ml fresh medium was replaced after removing the old medium and washing with 1 ml of PBS to remove the non-attached cells.

Refreshing the medium every three days about two wk, the bottom of culture flask was filled by cells. To avoid medium changing stress on the proliferation and cell growth, 1 mL of the previous medium was mixed with fresh medium during medium refreshment. Before 90% confluence of the cells, they were passaged. After, removing the culture medium and washing with PBS, the cells were trypsinized (1.5 ml 0.05% trypsin / EDTA, Sigma, USA) and incubated for 3-4 min to detach from the bottom of culture flask. Culture medium was added to the plate twice as much of trypsin added in the previous step to stop the enzyme activity. The cell suspension was collected in a Falcon tube and centrifuged at 1200 rpm for 10 min at room temperature. After pouring out the supernatant, 3 ml of the culture medium was added to the cell sediment and cells were re-suspended. In the next step, every 1 ml of this solution was added into 75T culture flasks that were already filled by 14 ml culture medium and were incubated. This process continued until cells reached to the third passage.

### Authentication of SCs' markers in the isolated BMSCs by reverse transcription-PCR

When SCs filled a T75 flask at the third passage, their culture medium was removed. Next, they were washed with 3 ml of PBS. After that, the cells were treated with 3 ml trypsin / EDTA for 3-4 min and after adding 6 ml of culture medium, centrifuged at 1200 rpm for 5 min. The resulting cell sediment was used for RT-PCR reaction. First, the entire contents of the RNA (Total RNA) were extracted using the extraction column kit (RNA Dena Zist Asia Company, Iran) according to the manufacturer's instructions. To ensure the quality of the extracted RNA, 5 ml of that was monitored over agarose gel electrophoresis as well as its absorption at a wavelength of 260 nm was evaluated.

The resulting RNA was kept in the freezer at -70°C until usage time. Next, the synthesis of complementary DNA (cDNA) was done using a kit (Accu power cycle script RT premix dN6, Bioneer, South Korea). According to the concentration of RNA, a decent amount of RNA was utilized in micro tubes, containing all the components needed to carry out the desired reaction. Then with the help of DEPC water, the reaction volume was adjusted to 20 ml. Reaction was carried out in a schedule with respect to time and temperature. The resultant product was kept in the freezer (at -20°C) until used. In the next step, polymerase chain reaction (PCR) was performed using specific primers (Table II). Mentioned reaction was done using master mix manufactured by Amplicon Company, so the necessary amount of cDNA (directed by the kit manufacturer) was added to micro tubes and placed in temperature and time process as follows. After the completion of the reaction in Veriti 96 well Thermal cycler (Applied Biosystems, USA), the presence of specific PCR products in desired size was assessed in 1.5% agarose gel under gel documentation system (UVTech Cambridge, UK) with the aid of DNA Safe Stain.

### Mating and pregnancy control

The vaginal smears was used to detect the stage of estrous cycle of female rats using microscopic observation (30). The female rats at proestrus or estrous stages were transferred to cage of a mature male rat and left overnight for mating. Thereafter, their early pregnancy was confirmed using the vaginal smear method (31). In detail, the presence of vaginal plug in the female rats were examined in the next morning. They were separated from the males. Cellular characteristics of vaginal smears were evaluated once again on days four and five post-coitus using light microscopic. A presence of cellular characteristics of diestrus or metestrus on days four and five post-coitus were the signs of a positive indication for pregnancy.

### Injecting ultra-fine glass needle preparation 

In order to make a simple, inexpensive, and relatively flawless DIY (Do-It-Yourself) needle for microinjection of cells in fetus, the following steps were performed.

#### Pulling the needle

**Figure 1:**
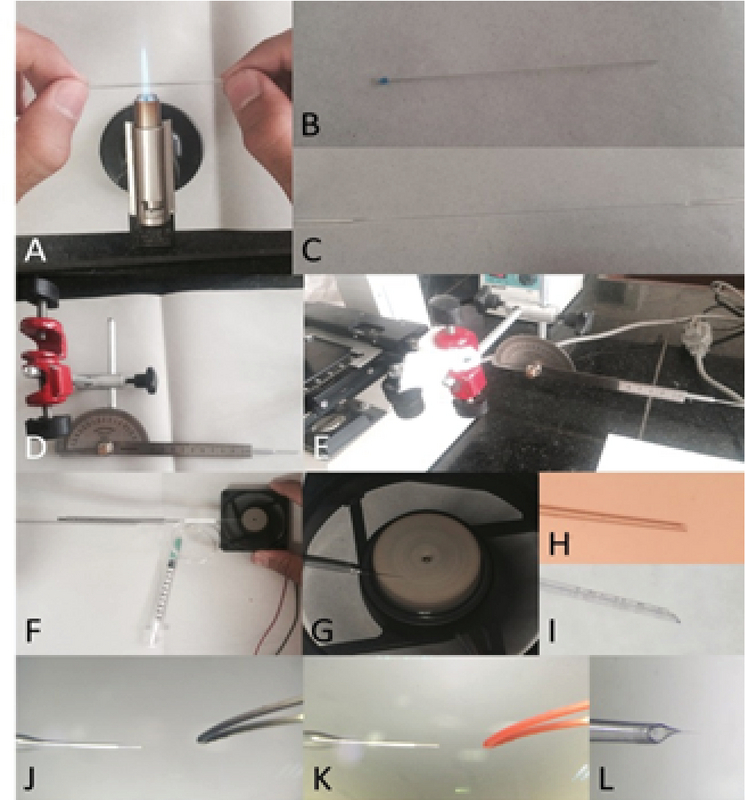
Steps involved in the preparation of a microinjection needle for the xenotransplantation of mice bone marrow-derived stromal/stem cells into a fetal rat liver. (A) To pull the capillary hematocrit tube on the flame, the two ends of the tube were kept by thumbs and index fingers and after melting the glass pulled it horizontally. (B) A capillary hematocrit tube before pulling. (C) A pulled hematocrit tube before breaking into two separate glass needles. (D) A micromanipulator structure containing two metal rods, a metal protractor, and a retractable antenna. (E) A micromanipulator attached to a stage of light microscope for fine moving in XYZ axes using coarse and fine adjustment knobs and stage control. (F) Attachment of pulled glass needle to the antenna of micromanipulator and a syringe-tube connection for pushing water into the needle. (G) Beveling the needle on an abrasive rotating disc. (H) A glassy fine needle before beveling. (I) A beveled fine needle. (J) A micro forge wire before heating. (K) Forging the tip of a needle by a hot nichrome wire. (L) A forged needle ready for microinjection.

A simple needle-puller was made manually. To do that, we found a torch lighter with a very sharp and focused flame (Figure 1A). We also used simple glass micro-hematocrit capillary tubes instead of sophisticated state-of-the-art capillary tubes (Figure 1B). To pull the capillary on the flame, we found a steady table to place the torch lighter on and kept the hands in a comfortable position. The two ends of the tube were kept by thumbs and index fingers in a way that they could be easily rolled between them. Then, the middle of the capillary was kept at the apex of the flame while rotating the capillary. As soon as the middle part of the capillary was soft, the tube was gently taken away from the flame and pulled horizontally as steady, fast, and far as it could be, before it hardened. Subsequently, the fine and filamentous glass that was keeping the two needles attached was broken (Figure 1C). Then, the two very sharp needles were beveled and forged. However, they can be used without any further modification; beveling and forging the needles can improve the results. Note that one needs to master the skill of pulling needles by hand through practice.

#### Micromanipulator and beveling the needle

The micromanipulator mechanism relies upon one important concept: a device that helps you move the tip of the needle, for instance, in a three-dimensional coordinate system with very high precision and preferably high accuracy. We could not afford a state-of-the-art and sophisticated micromanipulator. Thus, we made one for ourselves, putting to use a light microscope stage, two 20 cm-long metal rods, a metal protractor, and a retractable antenna (Figure 1D). Using a microscope stage, we were able to have enough precision for our work. We fixed one end of a metal rod on the microscope stage (Figure 1E). Next, we fixed the other end of this rod to one end of the other rod. Then, we attached the second rod and a protractor on its 90° line. Next, we attached the retractable antenna on the rotating guide of the protractor. The antenna works as the holder and the needle was installed on its tip. We made a simple and cheap needle beveller in our laboratory as well. First to make sure that glass dust does not clog our needle, we attached it to flexible tubing and a syringe full of deionized water (Figure 1F). Then an extra-fine abrasive disc was glued on a small 9 volt DC fan (a computer fan) and the fan was plugged to a power supply (Figure 1G). Then, the needle's tip was put upon the abrasive with a 30° slope (Figures 1H, 1I). As the needle was gently forced toward the abrasive, we pushed the water through the needle to drive the glass dust away.

#### Forging the needle

Moreover, we built a simple micro-forge. A short and fine nichrome (nickel and chromium alloy) heating filament was plugged into a 12-volt power supply (Figure 1J). Placing the filament under a stereomicroscope, we were able to sharpen the needles even further. We gently and steadily attached the very tip of our needles on the heated filament. After the tip of the beveled needle stuck to the filament, the power supply was turned off, and before the glass got hard again, it was pulled and sharpened (Figures 1K, 1L). The prepared microinjection needles were transferred to a box and sterilized in an autoclave machine.

### BMSCs preparation for injection

In order to inject BMSCs into pregnant rat fetal liver, the required amount of BMSCs from bone marrow labeled with eGFP was produced in the third passage. Then, 4 ml of medium culture was added to cells sediment and cells were suspended; 1 ml of this cells mixture was pulled into an insulin syringe, and thus, required cells were prepared for injection into the rat fetal liver. Before injection, a pulled needle was attached into a plastic tube of a 23-gauge butterfly needle after removing the needle part. Then the tube was attached to the filled syringe and cell suspension was gently pushed into the microinjection glass needle.

### Intrauterine BMSCs xenotransplantation 

This study was conducted on three pregnant rats at day 14 of gestational age that was determined through their vaginal plug (length of pregnancy in rats is approximately 21 days). These animals were provided from the Comparative and Experimental Medicine Center of Shiraz University of Medical Sciences, Shiraz, Iran. Pregnant rats were anesthetized with ketamine and xylazine. Animals were placed in the supine position and lower abdomen was shaved and then disinfected. Following this procedure, an incision was carried out in the lower abdomen vertically; after opening the fascia, subcutaneous layer, and muscles of that area, the uterus was removed from the abdomen and the position of embryos were checked. By applying pressure to the syringe, the cell suspension was gently forced into the pipette. A glass needle was entered into the amniotic fluid and then into the fetus in the area of the liver that was observed with microscope through the wall of uterus where each of the embryos were located. The injection of stem cells was carried out with 5 × 10${}^{6}$ cells in 200 µl culture medium in all fetuses. Next, the uterus was returned into abdomen. Following the stitch of abdominal cavity, the female rats were monitored for 24 hr in terms of behavior, health, eating, and risk sign of abortion.

### eGFP DNA tracing in rat liver by PCR

Two wk after the delivery, the newborn's liver as treated samples, mother's liver as negative control (and to ensure that there is no cellular migration), and mice liver as a positive control were removed. The sampling was done on livers and DNA was extracted using the tissue DNA extraction kit. Polymerase chain reaction (The PCR technique was used to study the presence of eGFP mice DNA. In brief, after adding some liquid nitrogen on it by the pestle, liver tissue was homogenized. Then, genomic material was extracted using a kit (AccuprepⓇ Genomic DNA Extraction Kit, Bioneer, South Korea). Next, using specific primers (Table II), the presence of *eGFP* gene in the resulting sample was evaluated through the PCR (Taq DNA Polymerase Master Mix Red, Amliqon, Denmark).

**Table 2:** The primer sequences used for quantitative real-time polymerase chain reaction (RT-PCR)


**Marker**	**Primers**	**Band size (bp)**
**CD34**	Forward-AATGAGTCTGTTGAGGAA Reverse-CTGTCTGAAGTAGTAGGC	215
**CD45 **	Forward-AAGTGGATGTCTATGGTTA Reverse-GAAGGAAGTCTCTGGTAT	226
**CD90**	Forward-GAAGACAAGGAGCCAGAAC Reverse-GCAAGGGAAAGAAGAATAAAGG	118
**eGFP**	Forward-GCACCATCTTCTTCAAGGACGAC Reverse-TCTTTGCTCAGGGCGGACTG	343

### Ethical consideration

The present experimental study was approved by the Shiraz University of Medical Sciences Ethics Committee (project number: 92-01-01-7021).

## 3. Results

### BMSCs culture findings

**Figure 2:**
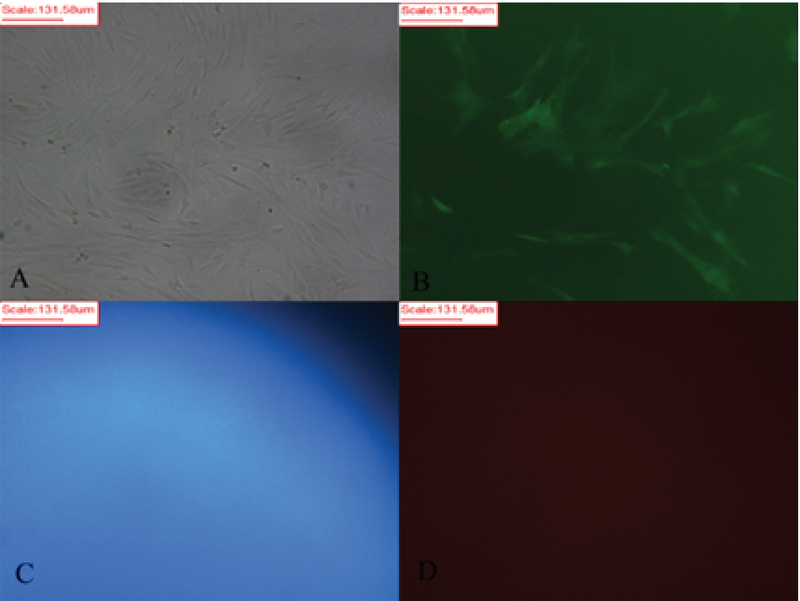
Green flurcent protein expression in mouse bone marrow-derived stromal/stem cells (BMSCs). (A) light inverted microscope. (B-D) The same field by epi-flurcent microscope (filters G, B, and R).

**Figure 3:**
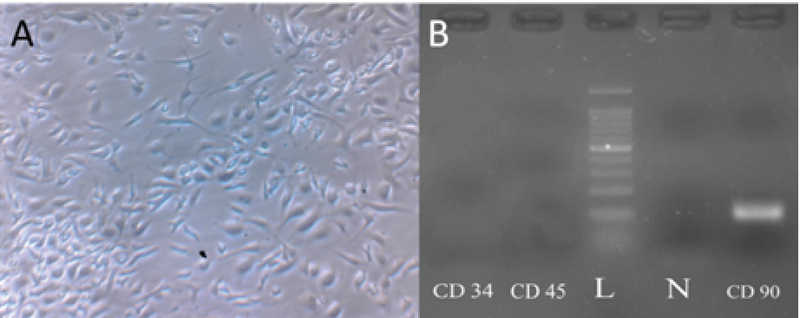
Characterization of mouse bone marrow-derived stromal/stem cells (BMSCs). (A) Fibroblast-like and spindle shape morphology of the mouse BMSCs, a typical characteristic of SCs in passage 3, 200x. (B) Agarose gel electrophoresis of products of reverse transcriptase polymerase chain reaction (RT-PCR) of BMSCs revealing to be positive for CD90 and absence negative for CD34 and CD45.

An important feature of mice used in this study was that they were transgenic so that due to the eGFP expressing gene insertion in their genetic material, most of the cells derived from these animals have been labeled and thus can easily be tracked, genetically. Inverted light microscope was used to image the isolated SCs (Figure 2A). The same field was also imaged by epi-fluorescent microscope with filter G (Figure 2B) that confirmed the expression of eGFP and filters B and R of the same field with now fluorescent signal to confirm the eGFP signal is not an autofluorescence signal (Figure 2C, 2D). In the early days of the primary culture, the population of BMSCs with spindle or fibroblast-like fetures had the highest number among others. Although rounded or flat cells were visible, too. Over time, the whole of culture medium was full of cells after about two wk. When the culture medium was filled with BMSCs, the cells were passaged; therefore, as a result of the treatment with trypsin/EDTA, the cells were separated from bottom of the culture plate and transferred to three T75 flasks. The rate of cell proliferation and growth were increased from passage one to next passages. Cultivation surface was filled out in about three to four days. The cells are often seen in the form of spindle or fibroblast-like fitures (Figure 3A). This process was continued until the third passage.

### BMSCs confirmation by SCs markers

In order to ensure the identity of the cells in the third passage, the presence of specific marker for SCs and the absence of specific markers of hematopoietic stem cells were evaluated. In this regard, the presence of CD90 mRNA (Thy-1 cell surface antigen) and the absence of specific mRNAs of CD34 (hematopoietic progenitor cell antigen) and CD45 (protein tyrosine phosphatase) were assessed (Figure 3B).

### Mice cells *eGFP* gene expression in rat livers

**Figure 4:**
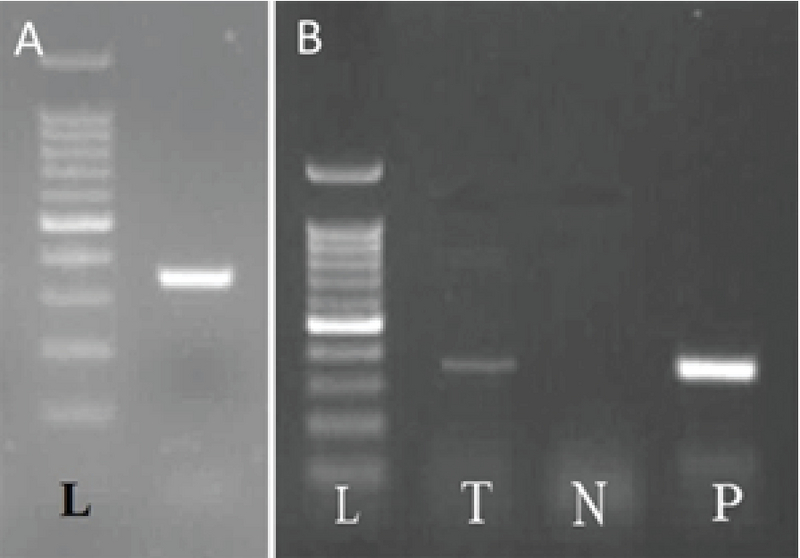
(A) Confirmation of *eGFP* gene expression in the liver of eGFP${}^{+/+}$ Balb/C mice by reverse transcriptase polymerase chain reaction (RT-PCR). (B) Confirmation of *eGFP* gene expression in liver of xenotransplanted rat livers. Rat infants 14 days after intrauterine mice eGFP-labelled bone marrow-derived stromal/stem cells (BMSCs) injection. T: treated injected rats; N: liver of negative control rat, mother of injected fetus; P: positive control, eGFP-labelled BMSCs.

By ensuring the proper functioning of primers of the *eGFP* gene (Figure 4A), the same process was performed on the liver of neonatal rats, and the presence of mice eGFP mRNA in the liver of in utero xenotransplanted rats were confirmed (Figure 4B). The same procedure was performed on the mother rat liver that confirmed the absence of the *eGFP* gene (Figure 4B).

## 4. Discussion

In the present study, the possibility of the presence and viability of mouse SCs in rats' infants after in utero transplant to fetal liver was measured. BMSCs obtained from mouse were prepared at the third passage. BMSCs are attractive alternative of successful stem cells-based treatments due to its availability, easy extraction method, and its rapid proliferation (32). In addition to the ability to differentiate into hepatic category (33), the SCs produce different cytokines and signaling molecules which can cause various effects in hepatic injury position such as immune response regulation, anti-inflammatory effects, anti-apoptotic and cell proliferation stimulation (34).

Intrauterine stem cell transplantation is considered as a medical procedure to treat a number of disorders and diseases and enjoys numerous advantages compared to postnatal treatment. For example, there is the ability to prevent progressive and irreversible damage to the fetus during the development (35). The foundation of stem cell transplantation to embryo is based on two assumptions: immature fetal immune system cells are not at a level of maturity in which it can distinguish between foreign and their own cells and therefore are not able to stimulate the appropriate immune response against the foreign cells. This eliminates the need of matching between donor and recipient MHC molecules. The high proliferative potential of the fetus environment in early pregnancy and normal migration of stem cells to different parts of body is the other benefit of this approach. Also, proliferating fetus is relatively empty of the presence of hematopoietic environment that, in turn, allows the establishment and transplantation of donor cells in large amounts, without having to discharge the host storage of hematopoietic (36).

However, further studies need to be done to understand the recruitment molecular mechanisms and deployment of SCs, growth, and proliferation in normal conditions of body or the ability to differentiate them into different cell lines in special environmental and developmental conditions. Also, it is necessary to identify the growth factors and effective chemokines involved in compliance and eventual transplantation of these cells in target tissues before applying at clinical level. Many factors, such as toxins, viral infections, immune system defects, and genetic disorders can lead to liver dysfunction and chronic or acute liver disease. In this situation, liver dysfunction can lead to conditions in which even the patient life is at risk. However, one unique characteristic of the liver is its ability to regenerate itself after injury. In response to simple liver damages like removing some part of liver tissue or after acute damages, restoration are carried out by proliferation of internal hepatocytes and without the need for auxiliary restorative processes. In chronic injuries in which the proliferation capacity of mature hepatocytes is inhibited, small, oval-shaped cells that express stem cell marker and biliary class cells marker, appear in the area surrounding liver tissue (37). It seems that these oval cells and precursor-like cells contribute to liver reconstruction by differentiating into hepatocytes (38).

In case of persistent injury, chronic disease and intensified liver fibrosis, the only effective treatment would be liver transplant method. However, the major limitation of this method is that usually there are not enough livers for transplantation. Also, side effects such as the lack of immunological compatibility and transplant rejection, irreversible complications associated with long-term use of medications that suppress the immune system, and the high cost of transplant surgery makes this method undesirable for many patients (39). However, on the other hand, the shortage of suitable organ for transplant will also lead to the death of many patients in transplant waiting list. It is therefore essential that new liver reconstruction methods be developed and the need for transplantation of some or all of liver tissue be met.

## 5. Conclusion

Despite the widespread use of BMSCs in clinical and pre-clinical trials of chronic liver disease, several subjects including the ability of differentiation and proliferation, the possibility of producing fibrous tissue, the best injection method, the best time stage of treatment, the most appropriate cell number, and frequency of injection should be considered. Therefore, the eGFP-genetically labeled BMSCs of adult mice were capable to remain functional probably as hepatocyte-like cells in the liver of infant rats after an in utero xenotransplantation. The therapeutic value of BMSCs lies in regulating the immune system and releasing the nutritious factors such as growth factors and cytokines.

##  Conflicts of Interest

The authors declare that there is no conflict of interests.
